# Nanoparticles as Alternatives for the Control of *Haemonchus contortus*: A Systematic Approach to Unveil New Anti-haemonchiasis Agents

**DOI:** 10.3389/fvets.2021.789977

**Published:** 2021-12-13

**Authors:** Rehman Ali, Nisar Ahmad, Sakina Mussarat, Abdul Majid, Sultan F. Alnomasy, Shahid Niaz Khan

**Affiliations:** ^1^Department of Zoology, Faculty of Biological Sciences, Kohat University of Science and Technology, Kohat, Pakistan; ^2^Department of Botanical and Environmental Sciences, Faculty of Biological Sciences, Kohat University of Science and Technology, Kohat, Pakistan; ^3^Department of Medical Laboratories Sciences, College of Applied Medical Sciences in Al-Quwayiyah, Shaqra University, Riyadh, Saudi Arabia

**Keywords:** *Haemonchus contortus*, nanoparticles, anthelmintic, gastrointestinal nematode, toxicity, anthelmintic resistance

## Abstract

*Haemonchus contortus* is an infectious gastrointestinal nematode parasite of small ruminants. This study addresses the *in vitro*/*in vivo* anti-haemonchiasis potential, toxicological effects, and mechanism of action of nanoparticles. Online databases were used to search and retrieve the published literature (2000 to 2021). A total of 18 articles were selected and reviewed, out of which, 13 (72.2%) studies reported *in vitro*, 9 (50.0%) *in vivo*, and 4 (22.2%) both *in vitro*/*in vivo* efficacy of different nanoparticles. Mostly, organic nanoparticles (77.7%) were used including polymeric (85.7%) and lipid nanoparticles (14.3%). The highest efficacy, *in vitro*, of 100% resulted from using encapsulated bromelain against eggs, larvae, and adult worm mortality at 4, 2, and 1 mg/ml, respectively. While *in vivo*, encapsulated *Eucalyptus staigeriana* oil reduced worm burden by 83.75% and encapsulated *Cymbopogon citratus* nano-emulsion by 83.1%. Encapsulated bromelain, encapsulated *Eucalyptus staigeriana* oil, and encapsulated *Cymbopogon citratus* nano-emulsion were safe and non-toxic *in vivo*. Encapsulated bromelain damaged the cuticle, caused paralysis, and death. Nanoparticles could be a potential source for developing novel anthelmintic drugs to overcome the emerging issue of anthelmintic resistance in *H. contortus*. Studies on molecular effects, toxicological consequences, and different pharmacological targets of nanoparticles are required in future research.

## Introduction

*Haemonchus contortus* is a highly infectious gastrointestinal parasitic nematode of small ruminants. The parasite causes acute anemia, hemorrhagic gastroenteritis, diarrhea, edema, stunted growth, and death of severely affected animals. *H. contortus* affects millions of ruminants annually, resulting in substantial economic losses due to decreased milk, meat, and wool production, loss of body weight, and cost of anthelmintic drugs (1, 2). The available anthelmintic agents such as imidazothiazole, benzimidazole, and ivermectin among others are becoming ineffective due to the rising issue of chemoresistance in helminths (3–7).

Helminth resistance to multiple anthelmintic drugs is increasing at an alarming speed and has raised great public health concerns (8). In the near future, it would be very difficult to control some of the parasites with prevailing anthelmintic drugs. Some studies reported that sheep nematode populations are highly resistant to oxfendazole (88%), levamisole (41%), and ivermectin (59%) in farm animals (9). Therefore, it is indispensable and timely to develop novel anthelmintics, which are suitable, environmentally friendly, cost effective, and potentially active.

Nanoparticles, owing to their small size, remarkable surface reactivity, and their biomedical applications, are becoming the leading candidates for the development of new anthelmintic drugs (10). They are able to cross membranes and generate reactive oxygen species (ROS), leading to great reactivity and finally death of infectious agents (11, 12). Recently, anthelmintic potential of nanoparticles is being constantly evaluated for controlling parasitic infections (13).

Nanoparticles are widely used in modern medicines, such as vaccines, diagnostic procedures, medical devices, drug delivery, imaging, and antimicrobial therapies (14). Several applications of nanomaterials as anthelmintics have been reported, including inorganic and organic nanoparticles (13, 15–29). Since the anthelminthic use of nanoparticles, we aimed to systematically address the *in vitro*/*in vivo* anti-haemonchiasis potential of nanoparticles, toxicological effects, and mechanism of action. This review will also help to highlight the existing gaps in nanoparticle research against *H. contortus*.

## Methodology

The systematic review was conducted according to the Preferred Reporting Items for Systematic Reviews and Meta-Analyses (PRISMA) guidelines (30). No protocol was followed for conducting this systematic review. The PRISMA checklist is provided in the supporting information section (Supplementary Table S1).

### Searching Criteria

Different databases, e.g., ScienceDirect, Google Scholar, Scopus, and PubMed were searched to find relevant published literature (2000 to 2021). Research articles published in the English language were gathered for this systematic review. Keywords such as nanoparticles, nanoparticles nematicidal activity, anthelmintic activity of nanomaterials, *in vitro*/*in vivo* activity of nanoparticles, and nanoparticles mechanism of toxicity/inhibition. “Nanoparticles AND anthelmintics OR nematicidal,” “Nanoparticles AND *Haemonchus contortus*,” “Anthelmintic AND *Haemonchus contortus*,” “anthelmintic *in vitro* OR *in vivo*.” The list of references of published articles was carefully observed, and related titles were searched and downloaded. Moreover, other related literature was also searched and included to discuss and support the findings of the current review.

### Inclusion/Exclusion Criteria

The inclusion criteria were (a) nanoparticles tested *in vitro*/*in vivo*, (b) articles containing information on assay types, concentration and time exposure used, inhibition/efficacy of nanoparticles, and size of nanoparticles, and (c) original research articles published in English. However, articles dealing with (a) molecular, prevalence, and epidemiological aspects of *H. contortus*, (b) nanoparticles studies dealing with parasites other than *H. contortus*, (c) studies that tested chemicals/drugs other than nanoparticles, (d) plant extracts used against *H. contortus*, and (e) nanoparticles used as a candidate for vaccine were out of the scope and were excluded from this review.

### Data Extraction

Endnote (Thomson Reuters, San Francisco, CA, USA) was used to compile the articles. The selected articles were carefully reviewed by the researchers to extract the relevant information including nanoparticle(s) name and size, biological species used, time of exposure, concentration used, inhibition/efficacy, toxicological and pharmacological effects, author(s) name, country of study, and year of publication. Figures and tables were formulated to arrange the extracted data. Moreover, Inkscape (0.92) (https://inkscape.org/) was also used as a drawing tool.

### Quantitative Analysis

#### Jaccard Similarity Index

Jaccard similarity index (JI) was calculated to determine the similarity between the two sets of studies reported in this review. One set of the study is the “*in vitro* pharmacological validation of nanoparticles” and the other one is the “*in vivo* pharmacological validation of nanoparticles.” The following formula was used for JI similarity (31):


$$\begin{array}{l} JI=c\times 100/\left(a+b-c\right) \end{array}$$

where “a” is the total number of nanoparticles used *in vitro*, “b” is the total number of nanoparticles used *in vivo* as anthelmintic against *H. contortus*, and “c” is the number of nanoparticles common to both *in vitro* and *in vivo* studies.

## Results

**Figure 1:**
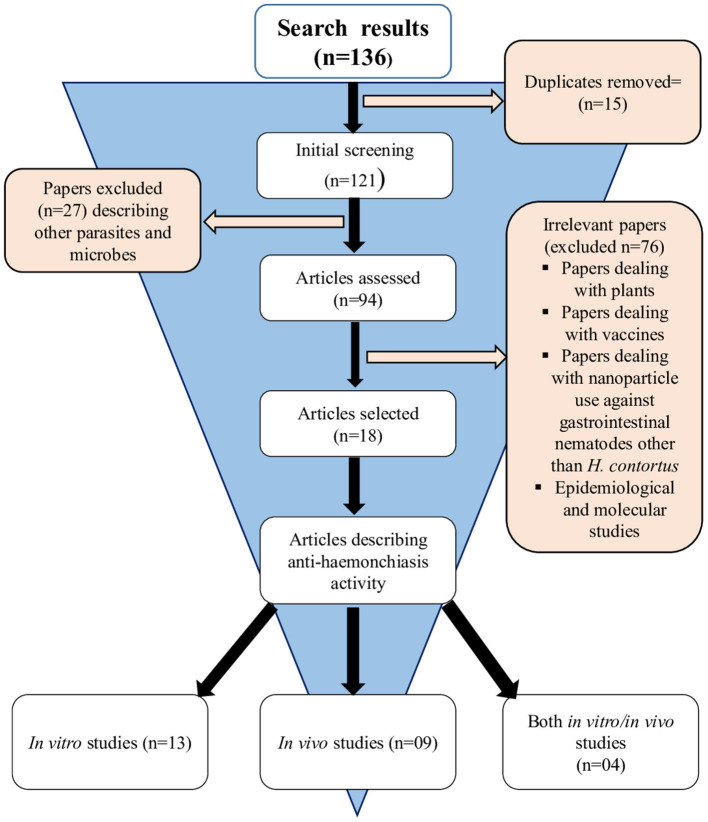
Article screening and selection process used for conducting this systematic review.

A total of 136 (*n* = 136) research articles were found and downloaded from online search databases. Eighteen (*n* = 18) articles were selected and thoroughly reviewed for this study. All the irrelevant and duplicate articles were removed (Figure 1). The quality of the selected articles was assessed, and the articles were summarized as author(s) name, nanoparticles used, biological species/compound used in combination with nanoparticles, source of nanoparticles, country name, release profile, reported quality control, as well as characterization (Table 1).

**Table 1:** Quality assessment of articles selected for this systematic review.

**Country Name**	**Type of nanoparticles**	**Biological specie**	**Release profile**	**Quality reported?**	**Nanoparticle's characterization?**	**Reference**
India	Silver nanoparticle (AgNPs)	*Ziziphus jujuba*	_	_	Transmission electron microscopy (TEM) and scanning electron microscope (SEM)	(20)
India	AgNPs	*Azadirachta indica*	_	_	TEM, and SEM	(22)
India	LAgNPs	*Lansium parasiticum*	_	+	Surface plasmon resonance (SPR)	(25)
China	Chitosan nanoparticles	Carvacrol and carvacryl acetate	+	+	Fourier transform infrared spectroscopy (FTIR)	(24)
Iran	Zinc oxide nanoparticle (ZnO-NPs)	N/A	_	+	XRD and TEM micrography	(13)
Brazil	Nanoemulsion	*Eucalyptus staigeriana*	_	+	Beam of red light (ZetaSizer 3600, Malvern, United Kingdom)	(29)
Brazil	Solid lipid nanoparticle	*Melaleuca alternifolia* (Maiden & Betche) Cheel	_	+	N/A	(19)
Brazil	Solid lipid nanoparticle	*Melaleuca alternifolia*	_	+	N/A	(18)
Brazil	Chitosan-encapsulated	*E. staigeriana* essential oil (EsEO)	+	+	N/A	(17)
Brazil	Nanoemulsion	*E. staigeriana*	_	+	Beam of red light (ZetaSizer 3600, Malvern, United Kingdom)	(21)
Brazil	Nanoencapsulated	*Eucalyptus citriodora* essential oils		+ _	FTIR analysis	(16)
Brazil	Encapsulated oil	*E. staigeriana*	_	+	N/A	(15)
Brazil	Nanoemulsion	*C. citratus* essential oil Nanoemulsion	_	+	Beam of red light (ZetaSizer 3600, Malvern, United Kingdom)	(23)
Brazil	Encapsulated oil	N/A	_	+	N/A	(27)
Brazil	Polycaprolactone Thio1 nanoparticles (nano Thio1)	*Tagetes patula* L.	_	+	Dynamic light scattering (DLS) (Zetasizer NanoZS™, Malvern Panalytical Instruments, UK)	(28)
Kenya	Chitosan encapsulated bromelain	N/A	_	+	SEM and FTIR analysis	(26)
Kenya	Chitosan encapsulated bromelain	N/A	_	+	FTIR analysis	(32)
Kenya	Encapsulated ethanolic extract	*Prosopis juliflora*	_	–	N/A	(33)

*Key: N/A, data not available*.

**Figure 2:**
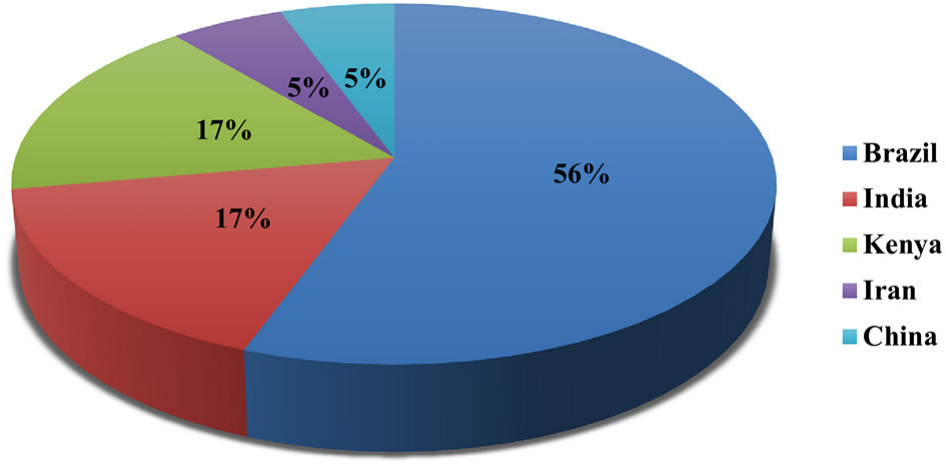
Country-wise studies of nanoparticles against *H. contortus*.

**Figure 3:**
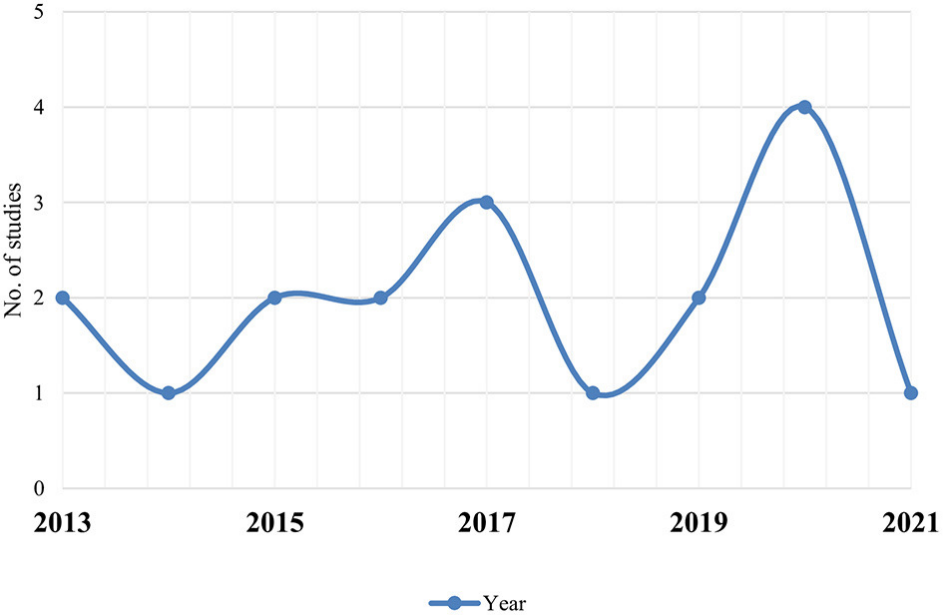
Year-wise studies of nanoparticles against *H. contortus*.

Out of 18 articles, 13 (72.2%) studies reported *in vitro*, 9 (50.0%) *in vivo*, and 4 (22.2%) reported both *in vitro*/*in vivo* efficacy of nanoparticles. *In vitro* studies were more than *in vivo*. Mostly, studies were carried out in Brazil (*n* = 10; 56.0%), Kenya, and India (*n* = 3; 17.0% each) (Figure 2). Most of the studies were reported in the year 2020 (*n* = 4) and 2017 (*n* = 3), followed by 2013 and 2016 (*n* = 2 each) (Figure 3). Mostly, organic nanoparticles (77.7%) were used including polymeric (85.7%) and lipid nanoparticles (14.3%). The remaining (22.2%) were non-organic nanoparticles comprised of metals (75.0%) and metal oxides (25.0%). Among metal and metal oxides nanoparticles, silver and zinc oxide were reported. The active substances were mainly encapsulated by using polycaprolactone and chitosan as a polymeric matrix. The release kinetics of chitosan encapsulated *Eucalyptus staigeriana* essential oil and chitosan nanoparticles loaded with carvacrol and carvacryl acetate were performed using dialysis membrane method. However, this important piece of information was found missing in most of the selected articles.

**Figure 4:**
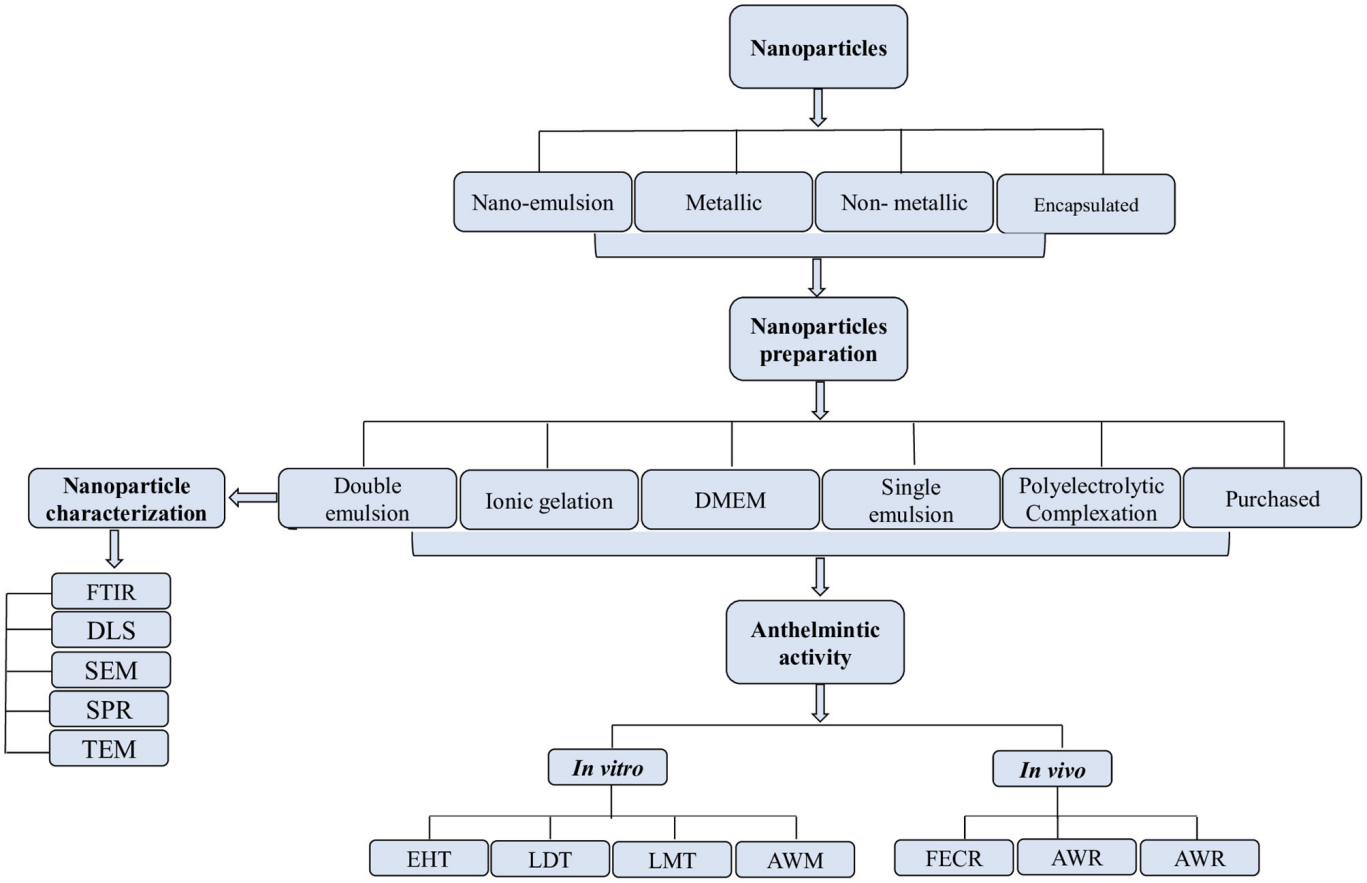
Process of selection, preparation, and characterization of nanoparticles for *in vitro*/*in vivo* anthelmintic activity.

The effects of nanoparticles were evaluated using the egg hatching test (EHT), larval development test (LDT), adult worm mortality test (AWM), and adult worm motility test (AWM) *in vitro*, whereas *in vivo* efficacy was evaluated by using worm burden reduction and fecal egg count reduction tests (FECRT). Egg hatching test was the commonly used assay *in vitro*, while the worm burden reduction was common *in vivo* (Figure 4).

The results exhibited that the doses used in the *in vitro* studies ranging from 0.025 to 56 and 0.20 to 500 mg/kg for *in vivo*. The most common exposure time against eggs hatching was 48 h, whereas it was 24 h for larvae and adults. The highest efficacy of 100% was a result of using encapsulated bromelain against eggs, larvae, and adult worm mortality at a concentration of 4, 2, and 1 mg/ml, respectively (Table 2). Encapsulated *E. staigeriana* oil reduced worm burden by 83.75% and encapsulated *Cymbopogon citratus* nano-emulsion by 83.1% *in vivo* (Table 3).

**Table 2:** *In vitro* efficacy of nanoparticles against *H. contortus*.

**Nanoparticle**	**Stage of parasite**	**Biological specie**	**Size (nm)**	**Concentration (mg/ml)**	**Time (h)**	**Efficacy (%)**	**Reference**
Silver nanoparticle (AgNPs)	Eggs Adult	*Ziziphus jujuba* Mill.	28–44	2 30	48 24	92 94	(20)
	Eggs Adult	*Azadirachta indica* A. Juss	15–25	0.025 0.025	48 24	85 87	(22)
	Eggs Larvae	*Lansium parasiticum*	300–700	15.8 nM 31.7 nM 63.5 nM 158.7 nM 15.8 nM 31.7 nM 63.5 nM 158.7 nM	48 24	32.1 45 47.2 51.2 33.3 29.5 22.2 14.8	(25)
Solid lipid nanoparticle	Egg Larval	(nanoTTO) Essential oil of *M. alternifolia*	N/A	0.1 0.2 0.4 0.85 1.7 3.5 3.5 7 14 28 56	24 48	2.77 3.50 15.22 21.19 41.52 82.63 19.51 40.63 48.73 67.28 84.80	(18)
Zinc oxide nanoparticle (ZnO-NPs)	Adult Adult	N/A	20–30	8 12 16 8 12 16 8 12 16 12 16 12 16	16 20 24 20 24	Low motility Very low No motility Very low No motility No motility No motility No motility No motility 19.33/20 20/20 20/20 20/20	(13)
Nanoemulsion chitosan	Eggs Larvae	*Eucalyptus staigeriana*	274	0.06 0.125 0.25 0.5 1 0.5 1 2 4 8	48 24	10.7 16.2 59.1 87.9 99 9.1 17.3 31.9 75.5 96.3	(29)
	Eggs	*Citriodora citratus* essential oil	248	0.07 0.15 0.31 0.62 1.25	N/A	34.9 49.4 58.1 73.3 97.1	(23)
Encapsulated bromelain (chitosan)	Eggs Larvae Adult	N/A	200–700	4 2 2	48 24 24	100	(26)
	Adult		N/A	1	N/A		(32)
Chitosan encapsulated EcEO	Eggs L1	*Eucalyptus citriodora* essential oil	N/A	0.125 0.25 0.5 1 2 4 0.5 1 2 4 8	N/A	11.9 25.9 56.8 85.5 92.8 100 10 39.1 49.4 75.8 98.0	(16)
Chitosan-encapsulated EsEO	Larvae Eggs	*E. staigeriana* essential oil (EsEO)	N/A	0.72 1.45 2.9 5.8 0.18 0.37 0.75 1.5		3.6 23.53 54.6 96.59 19.88 39.23 78.42 97.19	(17)
Chitosan nanoparticles	Adult	Carvacrol and carvacryl acetate	271–276	0.15	6 12	66.6 8.3	(24)
Encapsulated leaves ethanolic extract (ELEE)	Eggs	*Prosopis juliflora*	N/A	2	N/A	100	(33)
Encapsulated root ethanolic extract (EREE)						70	

*Keys: N/A, data not available; FECR, fecal egg count reduction*.

**Table 3:** *In vivo* efficacy of nanoparticles against *H. contortus*.

**Nanoparticle**	**Bioassay**	**Biological species**	**Size (nm)**	**Concentration (mg/kg)**	**Time (days)**	**Efficacy (%)**	**Model**	**Reference**
Nanoemulsion-chitosan EO	FECR Worm burden	*Cymbopogon citratus*	248	450	0 15	80 64 83.1	Sheep	(23)
Chitosan-encapsulated EO (EncEs)	Worm burden	*Eucalyptus staigeriana*	N/A	500	N/A	40.51	Mongolian gerbils	(17)
				365	30	83.75	Sheep	(15)
Solid lipid nanocarriers (nanoTTO)	Worm burden	*Melaleuca alternifolia*	287	0.20 0.50	N/A	4.09 48.64	Mongolian gerbils	(19)
Encapsulated bromelain	FECR	N/A	N/A	3 10 30	28	5 56.6 68.8	Goats	(32)
Chitosan encapsulated EcEO	FECR	*Eucalyptus citriodora*	N/A	250	10	40.5	Sheep	(16)
Encapsulated oils anethole + carvone	FECR	N/A	N/A	50	45	Significantly reduced FEC	Sheep	(27)
Polycaprolactone thio1 nanoparticles (nano thio1)	FECR	*Tagetes patula* L.	N/A	2,5	30	Kept the parasitic load stable	Sheep	(28)
	EPG			2.5	30	45%	Sheep	(28)
Nanoemulsion chitosan	EPG	*E. staigeriana*	277	0.25	180	No significant difference was observed	Sheep	(21)

*Key: N/A, data not available; EO, essential oil; nanoTTO, nano tea tree oil; FECR, fecal egg count reduction; EPG, eggs per gram of feces*.

The double emulsion method was the frequently used (*n* = 4) technique for nanoparticle preparation than the ionic gelation method (*n* = 2), and Dulbecco's modified eagle medium (DMEM), polyelectrolytic complexation system, and single emulsion method (*n* = 1 each). The methods used for nanoparticle characterization were Fourier transform infrared spectroscopy (FTIR), transmission electron microscopy (TEM), x-ray diffraction (XRD), and surface plasmon resonance (SPR).

### Comparative Analysis of Common Nanoparticles

Nanoparticles evaluated for both *in vitro* and *in vivo* efficacies were compared to know their effectiveness against the parasite. Four nanoparticles were commonly evaluated for *in vitro* as well as *in vivo* efficacy (Table 4). Since minimum concentration used and maximum efficacy obtained, the encapsulated bromelain was highly effective (100%) *in vitro*; however, the *in vivo* efficacy was not satisfactory (68.8%) at the tested concentration. Similarly, the chitosan-encapsulated EO (EncEs) were potentially active against eggs and larvae *in vitro* (98.0 and 97.0%, respectively), while high activity (84.0%) was also reported *in vivo* with a relatively higher concentration. Moreover, the nanoparticles were more effective *in vitro* compared with *in vivo*.

**Table 4:** Comparative analysis of common nanoparticles used against *H. contortus*.

**Nanoparticle**	***In vitro***		***In vivo***		**Toxicology**		**Mechanism of action**	**References**
	**Conc. (mg/ml)**	**Eff. (%)**	**Conc. (mg/kg)**	**Eff. (%)**	**Dose. (mg/ml)**	**T. level**		
Chitosan-encapsulated EO (EncEs)	5.8^b^ 1.5^a^	96.59 7.19	365^c^	83.75	500	Non	N/A	(17, 23)
Solid lipid nanocarriers (nanoTTO)	3.5^a^ 56^b^	82.63 84.80	0.50^c^	48.64	0.20	Non	N/A	(18, 19)
Encapsulated bromelain	1^c^ 2^b^ 4^a^	100	30^d^	68.8	3–30	Non	N/A	(26, 32)
Chitosan encapsulated EcEO	4^a^ 8^b^	100 98.0	250^d^	40.5	N/A	N/A	N/A	(16)

*Keys: a, eggs; b, larvae; c, adult worm; d, fecal egg count reduction; Conc., concentration; Eff., efficacy; T. level, toxicity level; EO, essential oil; TTO, tea tree oil; N/A, data not available*.

### Toxicity Evaluation

Toxicity and toxic doses of different nanoparticles were reviewed and reported (Table 5). Among the tested nanoparticles, nano-tea tree oil (TTO), EncEs, EcEOn, nanoencapsulated carvacryl acetate (nCVA), and encapsulated bromelain were reported as non-toxic at the tested concentrations, while AgNPs and EsNano were moderately and mildly toxic in HEK293 cell lines and female Swiss albino mice, respectively. Zinc oxide nanoparticles were not evaluated for their toxicity. Nanoparticles were either orally administered or through esophageal gavage. The LC_50_ for AgNPs, CcEOn, and nano-TTO was not calculated.

**Table 5:** Toxicity of nanoparticles used against *H. contortus*.

**Nanoparticles (symbol)**	**Concentration (mg/kg)**	**Exposure time (days)**	**Mode of administration**	**Model/cell line**	**Toxicity level**	**LC-_**50**_ value (mg/ml)**	**Physiological changes**	**References**
Silver nanoparticles (AgNPs)	31.7 nM 63.5 nM 158.7 nM	1	N/A	HEK293	Moderate	N/A	Viability of cell was decreased.	(25)
Solid lipid nanoparticles (nanoTTO)	0.20	5 7 9	Oral	Gerbils (*Meriones unguiculatus*)	Non	N/A	Non-toxic to liver and kidneys since hepatic and renal functions were not affected.	(18)
Nano emulsion *Eucalyptus staigeriana* (EsNano)	1,000 1,500 2,000 2,500 3,000	1–14	Esophageal gavage	Female Swiss albino mice (*Mus musculus*) Female Wistar albino rats	Mild	1,603.9	No significant differences were found in the body weights or the histological morphologies of organs between the treatment and control groups.	(29)
Nano-encapsulated EcEOn	2,000 2,500 3,000 3,500	15	Esophageal gavage	Female Swiss albino mice (*Mus musculus*)	Non	1,680.7	No behavioral changes and mortality were observed.	(16)
Nanoencapsulated carvacryl acetate (nCVA)	0.00156 0.3	1	N/A	Murine fibroblast L929	Non	0.3	No cytotoxic and genotoxic effects were observed.	(24)
Encapsulated bromelain	3–30	14	Oral	Goats	Non	0.155	No treatment related pathological changes of internal organs were observed after necropsy. No changes in the histology of heart, kidney, or hematology parameters were recorded.	(32)
Chitosan encapsulated *Eucalyptus staigeriana* EO (EnEsEO)	500	3	Oral	*M. unguiculatus*	Non	N/A	No hematological and biochemical alterations were reported.	(17)
Zinc oxide nanoparticles	N/A	N/A	N/A	N/A	N/A	N/A	N/A	(13)
*Cymbopogon citratus* EO nanoemulsion	450	3	Oral	Sheep	Toxic	N/A	One sheep out of ten died, treated with CcEOn. The sheep presented sialorrhea before death.	(23)
Encapsulated oils anethole + carvone	50	45	Oral	Sheep	Non	N/A	No effect on kidney and liver function.	(27)

*Key: N/A, data not available*.

### Jaccard Similarity Index

The two datasets, i.e., *in vitro* and *in vivo* use of nanoparticles was checked for their similarity by using JI similarity formula and 22.2% similarity was found.

## Discussion

The main constraints of profitable products in the livestock sector are parasites and parasitic resistance to anthelmintic drugs around the world. To resolve the huge economic losses, it is important to improve the control of main parasitic diseases through alternative, less harmful, biodegradable, and ecologically safe anthelmintic strategies. Nanoparticles may reduce the risk of resistance of *H. contortus* to the anthelmintic drugs and overcome the resistance mechanisms adapted by the parasite, potentiating the drug target, and increasing bioavailability of the drug. The current systematic review assessed the *in vitro*/*in vivo* nematicidal potential and toxicological implications along with the mechanism of action of various nanoparticles against *H. contortus*. The results of this study will help to identify potential approaches to design new nanoparticulate drugs and ways to meet the current research limitations in prospective studies.

Nanoparticles were commonly evaluated *in vitro*, and only few studies had reported *in vivo* effectiveness of different nanoparticles. Previously, *in vitro* studies were mostly reported than *in vivo* and justify the current findings (34–36). *In vitro* studies are inexpensive and less time consuming, and anthelmintic effects at different life stages of the parasite can easily be studied (37). After initial screening, effective substance/product can further be evaluated for *in vivo* efficacy (38). *In vivo* studies are useful to know the host immune response to a particular anthelmintic agent, toxicological and pharmacological effects, and *in vivo* efficacy. However, *in vivo* studies are expensive and difficult to reproduce the results, required long experimental duration, and has lower precision (39). The research field is highly inundated with *in vitro* studies, and *in vivo* studies are insufficient; therefore, *in vivo* evaluation of nanoparticles would be of great importance in future studies (36).

Organic nanoparticles were among the frequently used nanoparticles. Nanoparticles can easily be produced in large quantities using different approaches and are highly biodegradable and biocompatible. These nanoparticles possess the capacity to solve, absorb, and encapsulate a drug in a polymer matrix and are excellent nanocarriers for the controlled and sustained release of drugs (40, 41). Among metal and metal oxides, AgNPs and ZnO were reported. AgNPs have profound antiparasitic and antibacterial activities. Antiparasitic activity of AgNPs was inhibition of metabolic activities and cell proliferation of *Leishmania* spp. promastigotes. Antiviral activities of AgNPs have also been demonstrated to stop viral replication process and prevent binding of virus particles to host cell receptors. These nanoparticles have promising efficacy as anticancer agents and could be a reason that they have attracted more attention as an anthelmintic agent (42–46). ZnO nanoparticles are widely used and important candidates for developing novel drugs due to their non-toxic, antiparasitic, antifungal, and antimicrobial effects. These can also be used for gene delivery and can cause death of cancerous cells without effecting normal healthy cells (47, 48).

Nanoparticles were encapsulated using a polymeric matrix, mainly chitosan and polycaprolactone, to improve the controlled drug release. Encapsulation of bioactive substances also improves the absorption and bioavailability by facilitating the diffusion through epithelium. The most common are aliphatic polyesters and their copolymers. Polycaprolactone (PCL) is a synthetic aliphatic polyester approved by the FDA and has some advantages: it is hydrophobic, biodegradable, biocompatible, and relatively inexpensive (49). In addition, due to its low toxicity, it is suitable for intravenous or oral administration (50, 51). Chitosan, a natural polymer obtained by the deacetylation of chitin, was the frequently used encapsulating matrix. The chitosan microsphere formulation for the controlled release of drugs improves their dissolution and bioavailability (52, 53). Hence, increases in efficient drug delivery may increase the overall effectiveness of the targeted drug/compound. Furthermore, its excellent biodegradability and non-toxic nature were the core reasons for which chitosan was selected as an encapsulating agent for evaluating anti-haemonchiasis nanoparticles (21).

The release kinetics of nanoparticles was the neglected aspect and barely studied in the reviewed articles. It is an important and critical aspect that helps to understand the dosage form behavior, and assess the safety and efficacy of a desired drug during the various stages of development. To maximize the effectiveness of nanoparticle targeting, drug release from nanoparticles needs to be slow enough to avoid substantial drug loss before the carrier reaches the site of action thereby reducing toxicity (54, 55). After nanoparticle accumulation at the target site, optimizing efficacy will require tunability of the drug release rate (56). Therefore, determination of product quality and performance becomes a crucial aspect during nanoparticulate dosage form development. When designed appropriately, an *in vitro* release profile can reveal fundamental information on the dosage form and its behavior, as well as provide details on the release mechanism and kinetics, enabling a rational and scientific approach to drug product development (57). Thus, the kinetics of drug release from nanoparticles should be an essential feature of their design and a property monitored for the quality control of nanoparticle formulations (58).

The frequently reported assay was egg hatching test (EHT) followed by larval development test (LDT). The possible reason for such an extensive use of these assays may be attributed to the fact that these tests take into account variations in the habits, behavior, and sensibility of these life forms of the parasite and permit the exposure of different potential pharmacological sites for future pharmacodynamics investigation (59). Moreover, APMT and AWMT were less likely to be used in *in vitro* studies for anthelmintic evaluation. It is because of the lack of a culture system yielding adults of this nematode parasite, which prevents a preliminary investigation of the efficacy of anthelmintics at this stage (60); hence, the *in vitro* tests using free-living stages of nematode parasites are considered as the best means of screening the anthelmintic activity of new substances/products (61).

The most widely utilized *in vivo* assessment of nanoparticles against *H. contortus* was the FECR test. The major benefit of this examination is that, regardless of their mode of operation, it can be carried out with all anthelmintics (59). Gerbils and sheep have been primarily used in *in vivo* experiments as animal models. Using sheep as a model can be explained by the fact that domestic animals are a valuable component of clinical studies for several purposes, including simple availability and management, accessibility to early examine diseased tissues as well as the models, and also require disease characteristics to be explored at an early level (62). However, a study also evaluated the efficacy of plants against intestinal nematode parasites by using mice as models and reported high anthelmintic efficacy (63). Rodent use as an animal model may have some drawbacks; rodents provide a completely different internal environment (habitat) to the nematodes than small ruminants; thus, the drug efficacy may be lower or higher based on the habitat and drug absorption site of the host (64). Rodents are monogastric, and sheep are polygastric animals, which can also alter the drug mechanism of distribution and biotransformation. However, efficacy test on rodents can help researchers deduce the prescriptions to be used on sheep and goats (63).

### Toxicity

**Figure 5:**
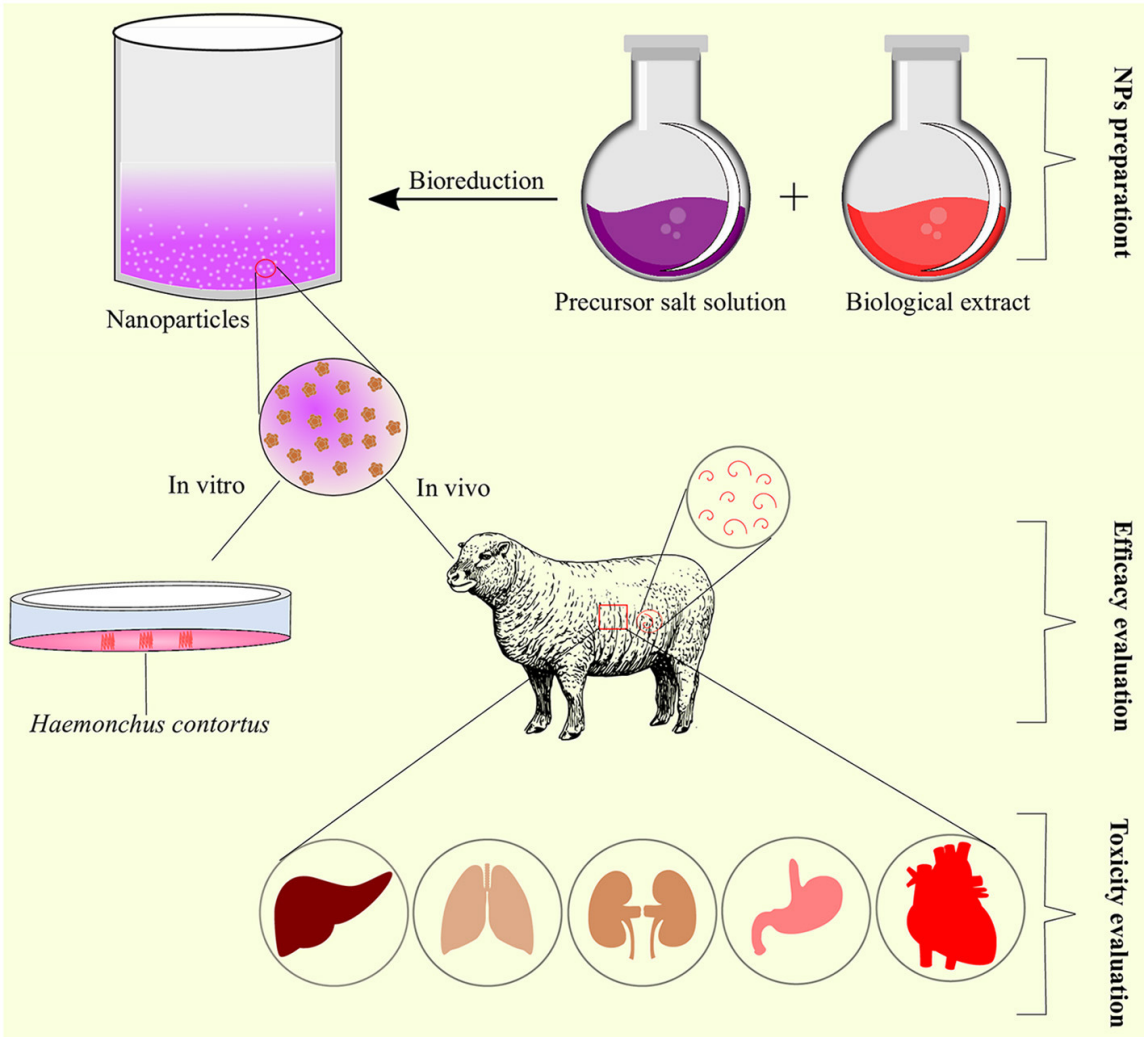
Schematic representation of nanoparticles preparation, efficacy, and toxicity evaluation as anthelmintic agents.

LAgNPs were moderately toxic when tested on HEK293 cell lines. The HEK293 cell viability decreased in a dose- and time-dependent manner. The lowest concentration produced no significant toxic effects; however, with an increase in concentration, i.e., 31.7, 63.5, and 158.7 nM, the observed decrease in the viability was 77.5 ± 5.06, 71.3 ± 9.8, and 62.1 ± 9.3%, respectively (25). Oral administration of CcEOn at 450 mg/kg concentration resulted in the death of 1 sheep out of 10. The sheep suffered from sialorrhea before death (23). However, the death of the sheep was not confirmed through necropsy and was assumed that the sheep may have aspirated the essential oil, and the wrong route of administration was attributed as the cause of death. Other studies also supported the non-toxic nature of CcEO at 1 and 800 mg/kg in rats and gerbils, respectively (65, 66) (Figure 5).

EsNano was found non-toxic after acute and subchronic toxicity evaluation in rats. The tested concentrations did not produce any change in the hematological parameters except a slight increase in the white blood cells (WBCs) after subchronic toxicity (29). There was no change in the body weight and histological morphologies of organs in treated rats (29). Nano encapsulated carvacryl acetate (nCVA) had no cytotoxic and genotoxic/mutagenic effects on murine fibroblast cell lines at the tested concentration. The non-toxic effects of nanoparticles were attributed to CVA and the biopolymers as they had no toxicity (24). Esophageal gavage administration of nanoencapsulated EcEO was safe, and no behavioral changes and mortality were recorded after acute toxicity evaluation (16). Similarly, nanoTTO produced non-significant differences in the hematological and serum biochemical profiles of the treated and untreated groups (18). Encapsulated bromelain was non-toxic as no pathological and histological changes were observed at concentrations ranging from 3 to 30 mg/kg after necropsy. The hematological parameters also remained unchanged at the same concentrations, confirming the non-toxic effect of bromelain *in vivo* (32). Encapsulated *E. staigeriana* essential oil (EnEsEO) was found non-toxic when orally administered to gerbils at 500 mg/kg concentration (17). However, the toxicity of ZnO-NPs was not evaluated and should be evaluated in future studies.

### Mechanism of Action

The pharmacological activity of a drug depends on how it interacts with the targeted biomolecules, i.e., receptors (67). Pharmacological activity is an important phenomenon to know the precise target of the nanoparticle with anthelmintic efficacy against the parasite or other organism/pathogen under observation (36).

Exposure of *H. contortus* to LAgNPs produced morphological and physiological effects. Morphologically, LAgNPs caused complete distortion of the cuticle and shrank the body. Physiologically, levels of reactive oxygen and nitrogen species were significantly increased, which resulted in oxidative stress and caused physical damage to tissues of the worm (25). In response to oxidative stress, a sharp increase in stress-responsive activities of enzymes, like catalase, superoxide dismutase, and glutathione peroxidase activities, along with the concentration of glutathione, was observed in worm tissue, which indicated a LAgNPs-responsive alteration of metabolism (25). Moreover, AgNPs also depleted the levels of glycogen, lipids, and protein contents of *H. contortus*. Parasites produce energy from stored carbohydrates (glycogen) to perform major metabolic processes (20). Glycogen is the chief energy reserve in most of the nematodes that exist in environments of low oxygen tension (68). Lipids are the chief functional and structural components of nematode parasites. Plasma membranes and eggs contain lipids as an important energy source in the free-living stages, any depletion or damage to lipid constituents may lead to mortality of the parasite. Therefore, lipid biosynthesis inhibition could be a potential target to develop an effective anti-haemonchiasis drug (20).

Proteins, like enzymes, are very important for normal physiological functioning and to carry out key metabolic activities. Hence, reduced protein content would hamper the normal physiological activities of the worms and may be accounted for mortality at higher concentrations. Egg morphological alterations justify the disintegration and shrinkage of *H. contortus* larvae development (20). Some studies reported, a drastic decrease in 5′ nucleosidase, ATPase, alkaline, and acid phosphatases of intestinal cestodes treated with AuNPs (69). Encapsulated bromelain is highly effective against nematode parasites (70), and it was found that bromelain damages the cuticle of *H. contortus* leading to paralysis and death (71).

The ZnO-NPs completely paralyzed the parasites. These nanoparticles can adversely affect the antioxidant systems of *H. contortus* by inducing severe oxidative stress resulting in denaturation of the antioxidant enzymes. Various concentrations of ZnO-NPs imposed controversial alteration on the activities of the antioxidant enzymes including superoxide dismutase (SOD), catalase (CAT), and glutathione peroxidase (GSH-Px) (13). This increase in the oxidative stress and reactive oxygen species (ROS) can damage proteins, carbohydrates, lipids, and DNA of the parasite (72). Therefore, disruption of the antioxidant system of the parasite unables *H. contortus* to survive against the host generated free radicals.

## Conclusion and Future Recommendations

Nanoparticles could be a potential source for developing novel anthelmintic drugs to overcome the emerging issue of anthelmintic resistance in *H. contortus*. Mostly, *in vitro* studies have reported the anthelmintic efficacy of nanoparticles. More studies are required to evaluate and describe the effects of nanoparticles on a molecular level, toxicological consequences, and different pharmacological targets along with exact mechanism of action using suitable animal models. Furthermore, the size of the nanoparticles was not determined in some of the studies, which is one of the crucial aspects of nanoparticles, and should be considered in future studies to provide more in-depth information of the nanoparticles under consideration.

Chitosan-encapsulated EO and encapsulated bromelain were highly effective both *in vitro* and *in vivo* with no observed toxic effects at the tested concentration. However, the release profile of mostly nano-encapsulated compound(s) was missing, and hence, the controlled and sustained drug release properties are unknown. These nanoparticles should further be evaluated and could be alternative sources of anti-haemonchiasis agents.

## Data Availability Statement

The original contributions presented in the study are included in the article/Supplementary Material, further inquiries can be directed to the corresponding authors.

## Author Contributions

RA and NA conceptualized the study, conducted the formal analysis, curated the data, and wrote the original draft. RA and SM formulated the methodology. RA sourced the software and provided supervision for the study. RA, SNK, AM, and SFA reviewed and edited the final manuscript. All authors contributed to the article and approved the submitted version.

## Conflict of Interest

The authors declare that the research was conducted in the absence of any commercial or financial relationships that could be construed as a potential conflict of interest.

## Publisher's Note

All claims expressed in this article are solely those of the authors and do not necessarily represent those of their affiliated organizations, or those of the publisher, the editors and the reviewers. Any product that may be evaluated in this article, or claim that may be made by its manufacturer, is not guaranteed or endorsed by the publisher.
